# Towards a petawatt-class few-cycle infrared laser system via dual-chirped optical parametric amplification

**DOI:** 10.1038/s41598-018-25783-0

**Published:** 2018-05-16

**Authors:** Yuxi Fu, Katsumi Midorikawa, Eiji J. Takahashi

**Affiliations:** Extreme Photonics Research Group, RIKEN Center for Advanced Photonics, RIKEN, 2-1 Hirosawa, Wako, Saitama 351-0198 Japan

## Abstract

Expansion of the wavelength range for an ultrafast laser is an important ingredient for extending its range of applications. Conventionally, optical parametric amplification (OPA) has been employed to expand the laser wavelength to the infrared (IR) region. However, the achievable pulse energy and peak power have been limited to the mJ and the GW level, respectively. A major difficulty in the further energy scaling of OPA results from a lack of suitable large nonlinear crystals. Here, we circumvent this difficulty by employing a dual-chirped optical parametric amplification (DC-OPA) scheme. We successfully generate a multi-TW IR femtosecond laser pulse with an energy of 100 mJ order, which is higher than that reported in previous works. We also obtain excellent energy scaling ability, ultrashort pulses, flexiable wavelength tunability, and high-energy stability, which prove that DC-OPA is a superior method for the energy scaling of IR pulses to the 10 J/PW level.

## Introduction

Since the invention of chirped pulse amplification (CPA) in 1985^1^, which overcame the obstacles in amplifying an ultrafast laser pulse without damaging optical elements or crystals in a beam line, ultrahigh-intensity laser systems have progressed rapidly. By combining a Ti:sapphire crystal and CPA, laser systems with pulse energies of 100 J order and pulse durations of 10 fs order and a PW-order peak power have been achieved with wavelengths near 0.8 *μ*m^2,3^. Owing to CPA technology, a new research field called *strong*-*field laser science* was born and has progressed rapidly^4–6^. Recently, strong-field laser science has been experiencing a shift towards the use of infrared (IR) driving wavelengths. This is because they enable the demonstration of *ponderomotive potential scaling* of the electron energy in a high-intensity laser field. The ponderomotive potential is given by *U*_p_[eV] = 9.337 × 10^−14^ *I* [W/cm^2^] (*λ*_0_[*μm*])^2^, which induces quiver motion in an electron from a laser field. This formula can be explained in terms of the so-called three-step model in a semiclassical framework^7^. The ponderomotive potential is proportional to the square of the laser wavelength (*λ*_0_), thereby a long-wavelength laser is suitable for scaling up the electron quiver energy independently of the laser intensity (*I*_0_). One application of the ponderomotive potential scaling is to increase the photon energy in high-order harmonic generation (HHG). Indeed, increasing the photon energy in HHG up to the “water window” region was demonstrated using a 1.5 *μ*m laser pulse in 2008^8^. From the viewpoint of ponderomotive potential scaling, ultrafast lasers with long wavelengths are highly required not only for the generation of fully coherent ultrashort x-ray sources^8–11^ but also for attosecond pulse generation with high-order harmonics, obtaining a deeper understanding of photoionization, the time-resolved probing of molecular structures, the study of ultrafast solid physics^5,12^, and so forth^13,14^.

Normally, optical parametric amplification (OPA), which is a wavelength conversion method, is employed to generate ultrafast IR pulses^15,16^. In OPA, a seed pulse is amplified by an ultrafast pump pulse in a nonlinear crystal through a parametric process. Owing to the limited sizes and damage thresholds of crystals, the output energy of an IR pulse via OPA is limited to the mJ level with pulse durations shorter than 50 fs^17^. To overcome the difficulty in the energy scaling of OPA, optical parametric chirped-pulse amplification (OPCPA), which employs a separate high-energy pump with a long pulse duration^18–20^, has been proposed and demonstrated. A high-energy pump pulse with a picosecond duration can be employed to avoid damaging nonlinear crystals in the OPA process. Up to now, the highest pulse energy ever reported for an IR OPCPA system has been 30 mJ with a pulse duration of 260 fs^21^. Note that the long pulse duration is due to the trade-off between efficiency and spectral bandwidth (a broader spectrum supports a shorter pulse duration and vice versa)^22^. In fact, a pump laser with a pulse duration of 1 ps level is required to efficiently generate an IR pulse with a broad spectral bandwidth^23^. Hence, the development of IR OPCPA strongly depends on the development of a high-quality ps pump laser.

Under the above circumstances, Takahashi *et al*. theoretically proposed a *dual*-*chirped optical parametric amplification* (DC-OPA)^24^ scheme for generating high-energy ultrashort IR pulses pumped by a Ti:sapphire CPA laser in 2011. By employing the DC-OPA method, we demonstrated a high conversion efficiency and a broad spectral bandwidth and obtained the highest pulse energy in the 1–2 *μ*m region with a pulse duration of shorter than 30 fs^25,26^. Since our demonstration^25,26^, DC-OPA has attracted interest from other researchers, who theoretically designed laser systems for generating TW-class sub-to-two-cycle IR fs laser pulses in the mid-IR (MIR) region^27,28^. DC-OPA technology clearly assists quantitative scaling in ponderomotive-potential-scaling experiments. For example, to scale up the output flux in HHG, the pulse energy of the driver laser becomes important. According to our previous energy-scaling experiment on HHG^8^, 100-mJ-class IR fs laser pulses enable the generation of sub-*μ*J soft x-ray pulses in the “water window” (2.3–4.4 nm) region, which are very useful for the biological imaging of living cells since the photons in the “water window” region are transparent to water but strongly absorbed by tissues. In addition, high-energy IR femtosecond pulses will be very helpful for the further energy scaling of isolated attosecond pulses (IAPs) by employing an IR two-color gating (IR-TCG)^29^ method for HHG. Furthermore, high-energy IR pulses also enable the production of ultrastrong light bullets with TW peak power over a distance of 100 m^30^.

In this work, using DC-OPA, we successfully generate IR pulses with a total energy of over 210 mJ, which is 1–2 orders higher than previously reported works using OPA and OPCPA with pulse durations shorter than 50 fs. A high conversion efficiency of over 30% is obtained, which is higher than that of IR OPCPA systems and comparable with that of a standard OPA system. Our energy-scaling experiment shows that DC-OPA has excellent energy-scaling ability without sacrificing high conversion efficiency. Flexible wavelength tunability over an octave spectral range within 1–3 *μ*m is obtained while the conversion efficiency is maintained. A short pulse duration of 44 fs (8.8 optical cycles) is achieved in our experiment. We also achieve a high energy stability (per shot) of ~1% RMS. We also find that the chirp relations between seed and pump pulses play an important role in obtaining high efficiency and a broad spectral bandwidth, in contrast to in standard OPCPA systems. By carefully controlling the chirps of the seed and pump pulses, the DC-OPA system can be optimized to operate with multiple outputs with different spectral bandwidths and temporal chirps. In a word, DC-OPA is capable of the further energy scaling of IR pulses to 1 J order and even 10-J-order while maintaining a broad spectral bandwidth with high efficiency. We demonstrate that DC-OPA is an ultimate method for providing ultrahigh-energy/peak-power IR femtosecond laser pulses.

## Results

### Breaking the bottleneck in energy scaling by conventional OPA

**Figure 1:**
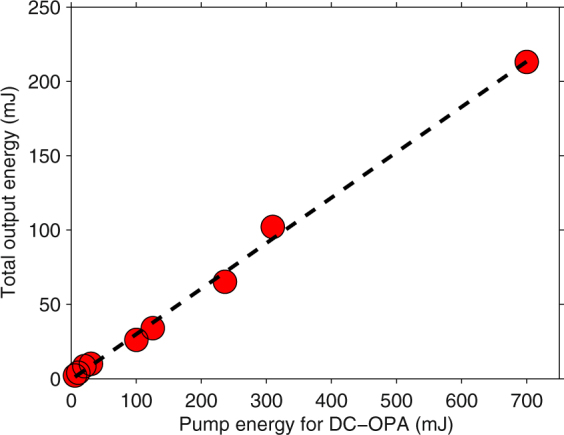
Energy scalability of DC-OPA. The central wavelength of the amplified signal pulse is tuned to 1.4 *μ*m.

As mentioned above, OPA is the most widely employed method for obtaining intense IR femtosecond laser pulses. However, the achievable pulse energy is limited by the aperture sizes of IR nonlinear crystals, which is due to the capability of crystal growth in manufacturing. The DC-OPA scheme can break this bottleneck as shown by our experimental result in Fig. 1. In contrast to conventional OPA, which allows a maximum pump energy of up to 10 mJ order, we can easily apply pump energy of more than one order higher in OPA using a commercially available crystal size (20 *mm* × 20 *mm* × *thickness* mm). The key technique^24^ is to increase the pump pulse duration when its pulse energy increases. In such a manner, the laser intensity on a *β*-BaB_2_O_4_ (BBO) crystal, which is proportional to the pulse energy and inversely proportional to the pulse duration and the square of the beam diameter, is kept below its damage threshold value. Meanwhile, the duration of the seed pulse is also temporally increased with the aim of obtaining an optimal temporal overlap with the pump pulse and achieving an efficient energy conversion. Since both the broadband pump and seed pulses are stretched (temporally chirped), the method was named dual-chirped optical parametric amplification^24^. Following the DC-OPA strategy, excellent energy scaling with 30.6% slope efficiency is obtained when the pump energy is varied from 6 mJ to 700 mJ with the pulse durations changed from ~25 fs to ~3.5 ps. Note that the necessary pulse duration also depends on the beam quality of pump pulse. If serious hot spots exist across the pump beam, its pulse duration must be further increased in the temporal domain to prevent the crystals from being damaged by these hot spots. For our pump laser, there are no serious hot spots. When the pump energy is 700 mJ, the total output energy is measured to be 213 mJ (signal: 128 mJ, idler: 85 mJ) and the measured spectra support transform-limited (TL) durations of ~40 fs. Owing to the energy limit of the pump pulse from our Ti:sapphire laser system, the maximum energy for pumping the DC-OPA system is 700 mJ. In fact, a DC-OPA system can accept a joule-class (100 TW to PW) pumping energy by further increasing its pulse duration^26^. In such a case, joule-class IR fs pulses with peak power approaching the PW class can be generated (see Table 1 and the Discussion section).

**Table 1:** Characteristics of OPA, OPCPA, FOPA, and DC-OPA for generating IR femtosecond laser in 1–2 *μ*m region.

	OPA^15–17,45^	OPCPA^18–20^	FOPA^46,48^	DC-OPA^25,26,36^
Pump source	fs Ti:sapphire CPA	ps laser (e.g. Yb:YAG)	fs Ti:sapphire CPA	fs Ti:sapphire CPA
Pump duration	TL	TL	Chirped	Chirped
Pump spectrum	Broad	Narrow	Broad	Broad
Maximum pump energy	0.1 J class	1 J	10 J order	100 J order
Seed duration	TL	Chirped	TL	Chirped
Seed spectrum	Broad	Broad	Broad	Broad
Synchronization	Automatic	Active stabilization	Automatic	Automatic
Energy scaling	Difficult	Easy	Possible	Easy
Output pulses	Signal and idler	Signal and idler	Signal	Signal and idler
Conversion efficiency	30–40%	10–30%	14%	30–40%
Highest reported output energy	10 mJ	30 mJ	30 mJ	100 mJ
Few-cycle pulse generation?	Yes	Yes	Yes	Yes
Preserve CEP stability of seed?	Yes	Possible	No	Possible
Wavelength tunability	Excellent	Good	Not reported	Excellent
How a broad output spectrum is efficiently generated.	Thin crystal with broad phase-matched bandwidth.	Thin crystal with broad phase-matched bandwidth. Pump duration on the 1 ps level.	Phase-matched bandwidth of crystal. Number of crystals in the Fourier plane.	Phase-matched bandwidth of crystal. Chirp management of pump and seed pulses.
Compressor	Not needed	Needed	Not needed	Most often but not always needed (See discussion of idler pulses in Fig. 4(b)).
Future energy scaling	0.1 J order	Depends on pump laser	1 J order	10 J order

### Broad wavelength tunability of DC-OPA

**Figure 2:**
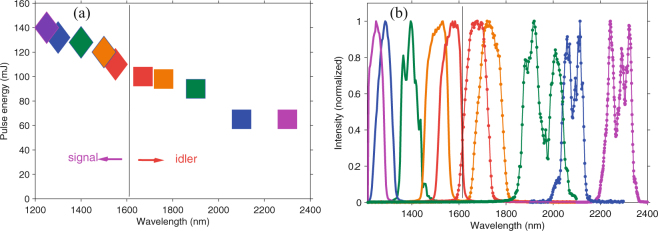
(**a**) Pulse energy and (**b**) spectra at different wavelengths. The pump energy for the DC-OPA system is 700 mJ.

The wavelength tunability of DC-OPA is confirmed in the next experiment, which is an important feature for extending the applications of a laser source. The experimental results are shown in Fig. 2, which shows a flexible wavelength-tuning capability in an one-octave spectral range of 1.2–2.4 *μ*m. The total conversion efficiency of DC-OPA is maintained at ~30% at each wavelength as can be seen in Fig. 2(a). Owing to the high conversion efficiency, the pulse energies of the signal beams remain above 100 mJ in the entire tuning range. In contrast, the corresponding idler pulse energies are measured to be 100 mJ or less. This is because a longer-wavelength pulse has a lower pulse energy owing to its lower photon energy under a constant conversion efficiency. Moreover, the pulse energy is clearly lower at 2.1 *μ*m and 2.3 *μ*m, which is attributed to absorption by BBO crystals at wavelengths beyond 2 *μ*m. The spectrum at each central wavelength is shown in Fig. 2(b). The spectral bandwidths at all these wavelengths support TL pulses of shorter than 10 optical cycles (FWHM).

### Optimization and characterization: the output performance of DC-OPA

In this section, the optimization and characteristics of DC-OPA are demonstrated and discussed, respectively. For clarity, the signal wavelength is set at 1.5 *μ*m unless specified.

#### High efficiency and broadband spectrum

In the first stage of the DC-OPA system (see Methods section), the seed pulse is amplified to 6 mJ, corresponding to a parametric gain of ~3000. In the second stage, the signal pulse is further amplified to 120 mJ with a gain of 20. No fluorescence is observed from the BBO crystals when the seed pulse is blocked. Under the optimized conditions for a signal wavelength of 1.5 *μ*m, the total output energy after the DC-OPA system is 218 mJ, which indicates an overall conversion efficiency of 31%. Considering only the second stage, the conversion efficiency is as high as 35%. Since our BBO crystals do not have any anti-reflection coatings, the conversion efficiencies of the whole DC-OPA system and the second stage are 34% and 38%, respectively, if the reflection of the pump pulse (~8%) on the crystal surfaces is taken into account. This high efficiency is similar to that of standard OPA^31^, which utilizes pump and seed pulses with a close to TL duration of 10 fs order, and is higher than that of OPCPA schemes. Hence, the DC-OPA scheme is a highly efficient method with an excellent energy-scaling capability for obtaining high-energy IR femtosecond pulses.

**Figure 3:**
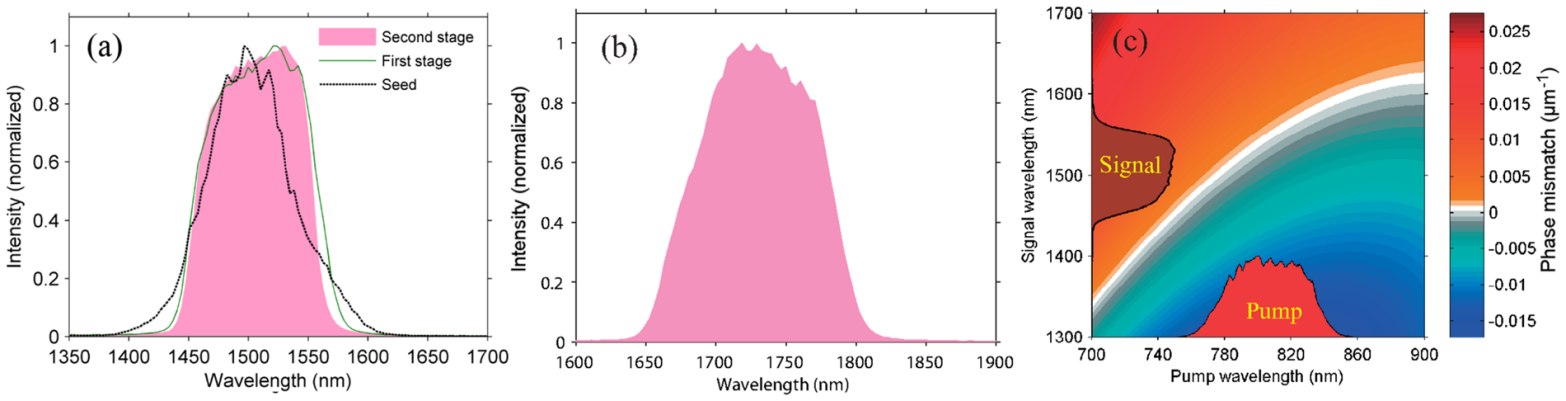
Spectra of (**a**) 100-mJ-class signal (1500 nm) and (**b**) idler (1730 nm) pulses after the DC-OPA system. (**c**) Calculated phase mismatch (unit of the color bar: *μ*m^−1^) between pump (red-shaded) and amplified signal (brown-shaded) pulses with different wavelengths across their spectra.

In conventional OPA, the temporal walk-off (group velocity mismatch) between signal and idler pulses in the parametric process limits the output spectrum bandwidth, resulting in a trade-off between efficiency and spectral bandwidth. The walk-off between signal and idler pulses generally becomes comparable to their pulse durations after passing through a BBO crystal of mm-order thickness. In contrast, the output spectral bandwidth of DC-OPA is not restricted by the temporal walk-off between signal and idler pulses. In our experiment, the walk-off is less than 5% of the pulse duration, which can be neglected. The pink-shaded regions in Fig. 3(a,b) show the spectra of 100-mJ-class signal and idler pulses, respectively. Their spectra are sufficiently broad to support TL pulse durations of ~40 fs and ~50 fs, respectively, which contain ~8 optical cycles. In the DC-OPA scheme, the spectral bandwidth is determined by the instantaneous phase matching^32,33^ and is affected by the seed-to-pump duration ratio. This is why the spectral bandwidths of the amplified signal pulses after the first and second stages are similar but narrower than that of the seed pulse in Fig. 3(a). To obtain a better understanding of the instantaneous phase-matching effect, we calculate the phase mismatch (*μ*m^−1^) between he pump and signal pulses with different wavelengths across their spectra. The result is shown as the two-dimensional plot in Fig. 3(c). The white-to-gray colors show the region with perfect phase matching. The spectra of the pump (red) and amplified signal (brown) pulses are also depicted. It is clear that the entire spectrum of the pump pulse is phase-matched to generate a broad spectrum of the signal pulse that follows the phase-matched curve. Thus, it can be concluded that further energy scaling using our DC-OPA system will maintain the spectrum bandwidth, which resolves the long standing difficulty of the trade-off between the spectrum bandwidth and efficiency in OPA. On the other hand, the reason why the signal pulse has a narrower spectral bandwidth than the seed pulse is that the spectral range of our pump laser is not sufficient to phase-match the entire spectral range of the seed pulse. Further increasing of the spectral bandwidth after the DC-OPA system will be discussed later in this paper.

From Fig. 3(c), it is also clear that when the pump and signal (with the same chirp as the seed) pulses have the same chirp sign, optimum phase matching can be achieved across their spectra in our experiment. In the following section, we will confirm this effect experimentally by precisely tuning the dispersion of seed pulses.

#### Flexible control of spectral bandwidth and temporal chirp

To investigate the chirp effect, we vary the temporal chirp of the seed pulse by tuning its GDD and TOD values. The GDD and TOD values of a 530 mJ pump pulse are set at ~25000 fs^2^ and −48000 fs^3^, respectively, which indicate a pulse duration of ~2.9 ps.

**Figure 4:**
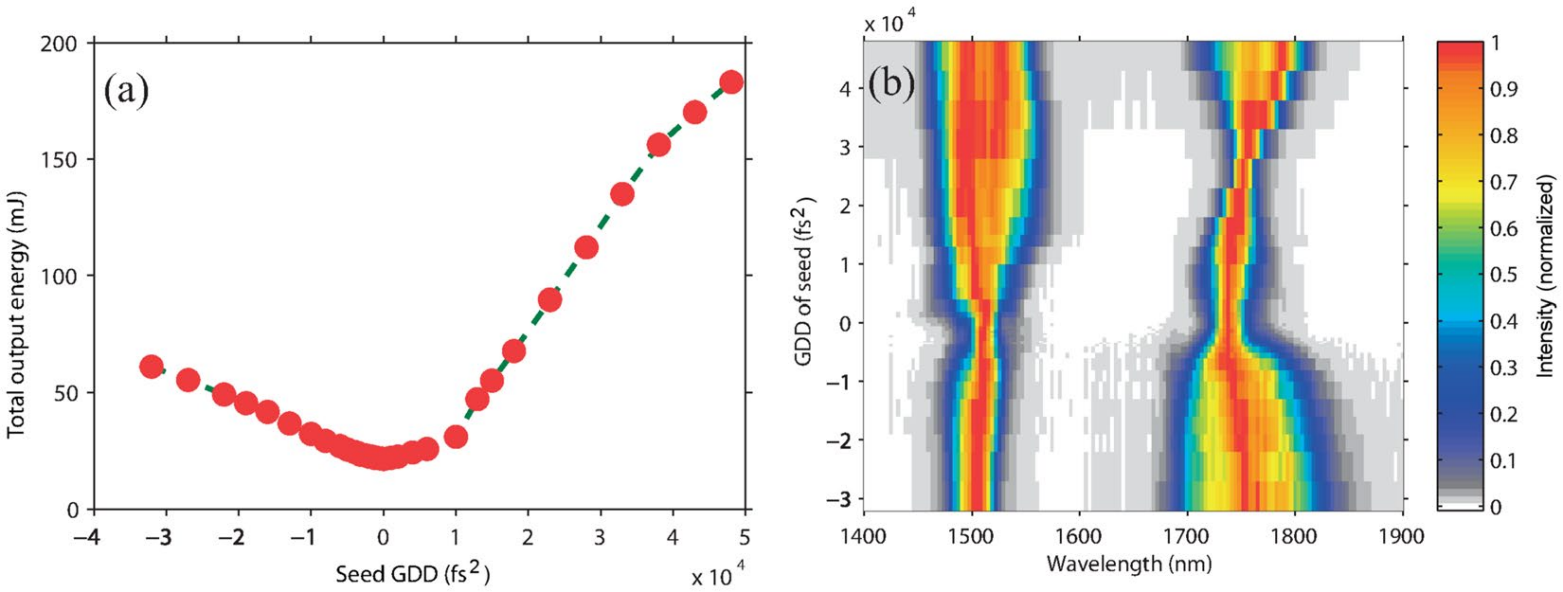
Dependences of (**a**) output energy and (**b**) spectra after DC-OPA on GDD of seed pulse with color scale indicating intensity (arb. units).

First, we only tune the GDD of a seed pulse, which affects the output energy as well as the spectra in DC-OPA as shown in Fig. 4(a,b), respectively. When the GDD of the seed pulse is set to 0 fs^2^, the output energy has a minimum value of 21.5 mJ. In this case, only a small portion of the pump pulse is temporally overlapped with the seed for the shortest pulse duration. For the same reason, the conversion efficiency increases when the seed pulse has a longer duration at larger absolute GDD values. Another notable feature is that the conversion efficiency is higher when the seed and pump pulses have the same sign of the GDD (positive). For example, the output energy is 112 mJ for a seed GDD of 30000 fs^2^ but only 61 mJ for the same GDD value with the opposite sign. The reason for this can be understood from Fig. 3(c). Better phase matching between different pairs of wavelengths of the seed and pump pulses that overlap at different time slices is achieved when the seed and pump pulses have the same sign of the GDD. This is an interesting feature in DC-OPA, that is not found in OPCPA. Note that the optimum chirp signs of the seed and pump pulses observed experimentally may vary with the laser wavelengths, the type of crystals and their cutting angles.

Figure 4(b) shows the spectra of the signal (1500 nm) and idler (1730 nm) pulses corresponding to Fig. 4(a). The spectra show a complex characteristic with respect to the GDD of the seed pulse. For the signal pulse, positive GDD values are more favorable than negative values for creating a broader spectrum bandwidth. This is because a better phase matching can be realized, as shown in Fig. 4(a). When the GDD of the seed pulse increases from 0 fs^2^ to 50000 fs^2^, there is an optimum GDD value near 40000 fs^2^ at which the signal pulse has the broadest spectral bandwidth. Thus, it is confirmed that the best phase-matching condition is achieved at different wavelengths across the spectra of the seed and pump pulses. The above features are discussed in more detail in the Supplementary Information (see Supplementary S-Fig. 1 and discussion). It can also be observed that the narrowest spectrum bandwidth appears when the GDD of the seed pulse is 0 fs^2^. This is because the signal pulse has the shortest duration. Then the temporal walk-off (group velocity mismatch) between the signal and idler pulses is comparable to their pulse durations. In other words, the walk-off becomes a dominant factor for determining the spectral bandwidth when the pulse duration is short, which is similar to in conventional OPA^15^. For an idler pulse that is generated through difference frequency generation (DFG) between the pump and seed pulses, its spectral bandwidth is mainly determined by the frequency difference range between the temporally overlapped seed and pump pulses. When the GDD values of the pump (positive) and seed (negative) pulses have the opposite chirp sign, long-wavelength components of the seed pulse overlap with short-wavelength components of the pump pulse in the temporal domain and vice versa. Hence, the DFG between the pump and seed pulses has a broader bandwidth^24^. In contrast, the idler pulse has the narrowest bandwidth when the seed GDD is near 26000 fs^2^, where the frequency differences between the wavelengths of the pump and seed pulses that are temporally overlapped is small. In other words, the narrowest spectrum of the idler pulse is because the chirp rates of the seed and pump pulses are similar. Thus, it is expected that the idler pulse is almost chirp free even without compression. This is because chirps of the seed and pump pulses nearly cancel out in the DFG process.

**Figure 5:**
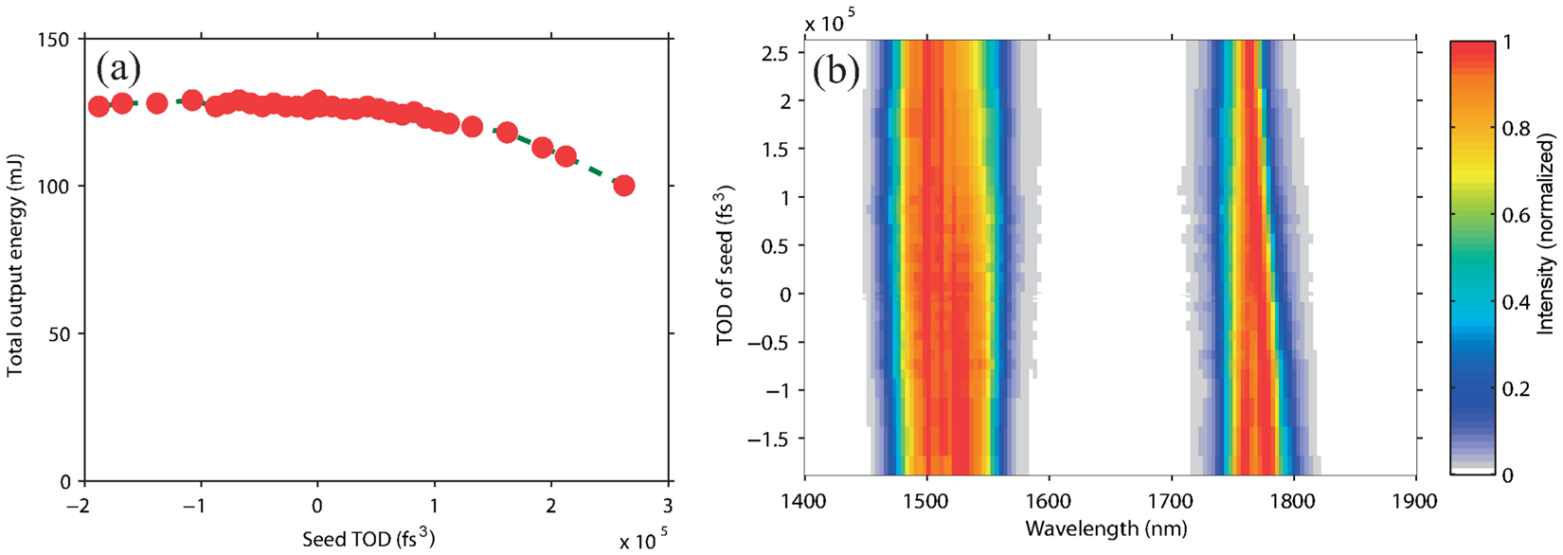
Dependences of (**a**) output energy and (**b**) spectra after DC-OPA on TOD of seed pulse with color scale indicating intensity (arb. units).

Next, we investigate the effect of the TOD when the GDD of the seed pulse is set at 36000 fs^2^. The experimental result is shown in Fig. 5. As the TOD of the seed pulse changes from negative to positive, the conversion efficiency slightly decreases as shown in Fig. 5(a). However, the dependence of the output energy on the TOD is much weaker than that on the GDD even though a very wide range of TOD values from −2 × 10^5^ fs^3^ to 2.5 × 10^5^ fs^3^ are assigned to the seed pulse. This is because the TOD does not significantly change the duration of a seed pulse as the GDD does. Thus, the seed pulse is still well overlapped with the pump pulse when different TOD values are assigned to the seed pulse in the experiment. This is also why the spectral bandwidth of the signal pulse does not change significantly with the TOD value as shown in Fig. 5(b). For the idler spectrum, there is an observable change with the TOD value. When the seed and pump pulses have a TOD with the same sign, the idler pulse has a slightly broader spectral bandwidth. This is because the spectrum of the idler pulse is determined by the temporally overlapped spectral components of both the seed and pump pulses. When the TOD of the seed pulse changes, the wavelength components across its spectrum are slightly modified in time domain. Hence, there is a slight change in the spectrum as the TOD change. A further discussion is given in the Supplementary Information (see Supplementary S-Fig. 2 and discussion).

From these experiments, it is clear that a high-energy broadband signal pulse can be obtained if different wavelength components of the pump and seed pulses are phase-matched with each other when broadband seed and pump pulses are employed for DC-OPA. Moreover, it is very interesting that DC-OPA can be operated in different modes that generate IR pulses with different spectral bandwidths and temporal chirps. For example, a broad-bandwidth idler pulse with a duration of a few cycles can be generated using chirped seed and pump pulses with opposite chirp signs. Since the idler pulse is generated by DFG, its carrier-envelope phase (CEP) is passively stabilized. In addition, a narrow-bandwidth and chirp-free idler pulse^24^ can be obtained when seed and pump pulses are chirped at the same rate, which is very useful for obtaining high-energy narrow-band laser pulses without phase compensation. These narrow-band pulses are useful for stimulated Raman researches or pumping FIR OPA systems^28^.

#### Characteristics of temporal pulse duration, energy stability, and beam quality

**Figure 6:**
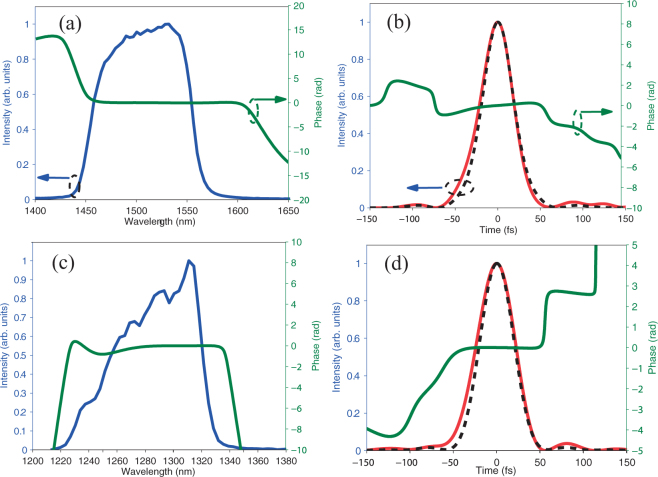
Pulse durations measured by SPIDER. Spectra (blue solid line) and phases (green solid line) of a compressed pulse at 1500 nm and 1300 nm are shown in (**a**,**c**), respectively. Reconstructed pulse envelopes (red solid line) and temporal phases (green solid line) are shown in (**b**,**d**), respectively. TL pulse envelopes are shown by black dashed lines.

When the pump energy is ~700 mJ with the pulse duration increased to ~3.5 ps and the GDD is ~30000 fs^2^, we obtain energies of 120 mJ (signal, 1500 nm) and 98 mJ (idler, 1730 nm) as shown in Fig. 2(a). Under these conditions, the seed pulse is stretched to ~3.7 ps with a GDD of ~50000 fs^2^. The high-energy signal pulses with positive chirp are compressed by a prism compressor consisting of two large Brewster angle cut prisms (SK1300). To avoid damage or self-phase modulation inside the compressor, the beam size is increased to ~50 mm. The prism has high transmission for p-polarized pulses under Brewster angle incidence. Thus, the prism compressor can have a throughput of over 85% in our experiment. The calculated phase of the prism compressor with the opposite sign is applied to seed pulses using an acousto-optic programmable dispersive filter (AOPDF). The pulse is compressed to a near-TL duration by further precisely optimizing the spectral phase of the seed pulse. We employ spectral phase interferometry for direct electric-field reconstruction (SPIDER) to characterize the compressed pulse, which is shown in Fig. 6. Figure 6(a) shows the spectrum (blue solid line) and the measured phase (green solid line). The reconstructed temporal pulse (red solid line) and phase (green solid line) are plotted in Fig. 6(b). The pulse duration is evaluated to be 44 fs (FWHM) and consists of 8.8 optical cycles, which is close to its TL duration (black dashed line) of 41 fs. Furthermore, we give another example of compressing pulses at 1300 nm using the same compressor. Owing to the difference in the dispersion of the prism compressor between 1300 nm and 1500 nm, the separation between the two prisms is increased. The spectrum (blue solid line) and measured phase (green solid line) are shown in Fig. 6(c). The pulse is compressed to 49 fs (FWHM), with its temporal phase shown by the green solid line in Fig. 6(d). The TL duration of the pulse is 45 fs (FWHM) as shown by the black dashed line. In fact, for the full spectral tuning range of the signal pulses of 1.2–1.6 *μ*m, we are able to compress the pulse duration using the same prism compressor. Hence, we can conveniently tune the central wavelengths of compressed pulses.

For the idler pulses, their chirps are determined by the chirps of both the pump and seed pulses. In our experiment, the idler pulses have negative chirps. By using an Öffner stretcher, which can provide positive chirps for laser pulses, the idler pulse can be compressed^34,35^. The shape of the idler pulse can be indirectly controlled precisely by adjusting the seed phase using an AOPDF, which is helpful for compressing its pulse to the TL duration^36^, especially for a few-cycle duration. Note that the chirp of idler pulses is determined by the operating mode of DC-OPA, as shown in Figs 4 and 5 and discussed above. Different types of compressors are needed or not necessary to compress idler pulses, which may have positive, negative, or zero chirps.

**Figure 7:**
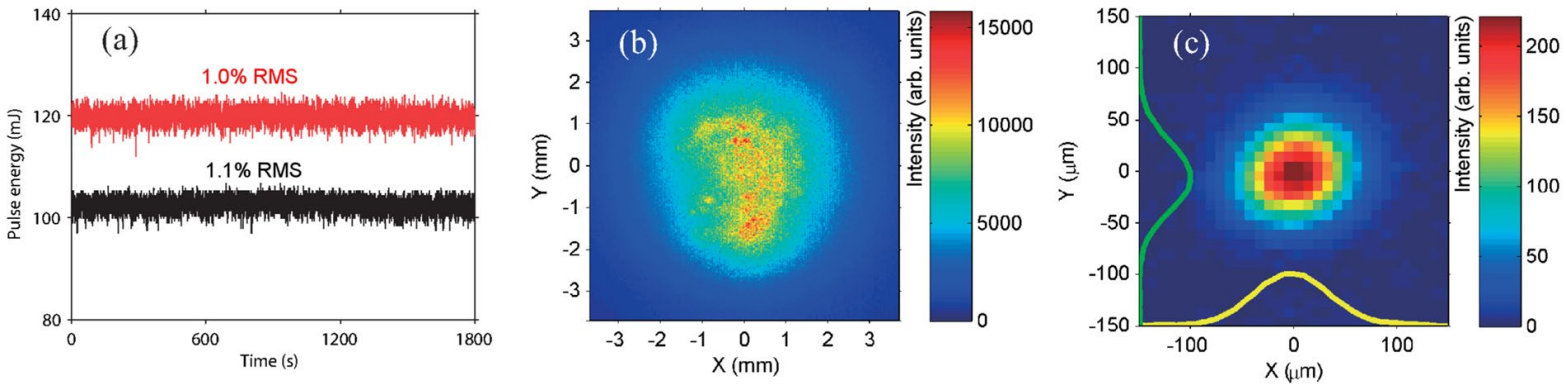
(**a**) Shot-to-shot energy stabilities of signal (red, 1500 nm) and idler (black, 1730 nm) pulses. Beam profiles of a 1.5 *μ*m pulse at the near field (**b**) and focus (**c**).

We evaluate the shot-to-shot pulse energy of signal (1.5 *μ*m) and idler (1.73 *μ*m) pulses. The results are shown in Fig. 7(a). Over 30 min, the energy fluctuations are 1.0% RMS and 1.1% RMS for the signal and idler pulses, respectively. High stability is achieved because the DC-OPA system is operated near a saturation condition.

The near-field beam profile of the 1.5 *μ*m signal pulses after the prism compressor is measured by an IR CCD camera (XC403, Xenics). Since the beam is too large for the IR camera to measure, we down-collimate the beam size to ~8 mm. The measured beam profile is shown in Fig. 7(b). We also check the focusability using an f/38 focusing geometry. The measured beam profile is shown in Fig. 7(c), and has a smooth intensity distribution. The focus size is measured to be ~140 *μ*m (1/*e*^2^). This focusability is sufficient for strong-field physics research such as on HHG.

## Discussion

To obtain deeper insights into the features discussed in the previous section, advantages and future prospects of the DC-OPA scheme, we give a detailed discussion in this section.

### Generation of high-energy, few-cycle IR pulses by DC-OPA

**Figure 8:**
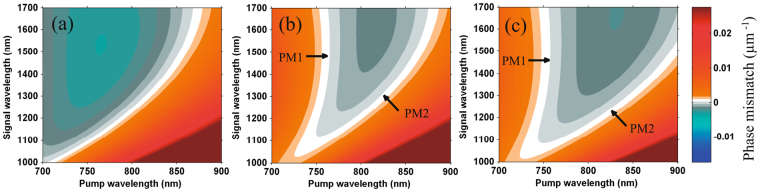
Phase mismatch (unit of the color bar: *μ*m^−1^) between different wavelength components across spectra of pump and signal pulses under type-I cutting of (**a**) BBO, (**b**) BiBO, and (**c**) YCOB crystals.

To achieve an even broader spectrum that supports a signal pulse with few-cycle TL duration (the seed spectrum should be sufficiently broad), two methods can be followed in DC-OPA. One method is to employ a pump laser with a broad spectrum^37–39^. The other method is to employ other crystals with a broader phase-matching bandwidth. Note that the energy-scaling ability of DC-OPA is not restricted by the dimensions and type of crystals. Here we show three examples of calculation results of phase-matching conditions using BBO, BiB_3_O_6_ (BiBO), and YCa_4_O (BO_3_)_3_ (YCOB) crystals with type-I cutting angles, as shown in Fig. 8. Pump and seed pulses propagate collinearly, by which the angular dispersion of the idler pulses can be avoided. In Fig. 8(a), signal pulse with a broad spectrum of 1.1–1.7 *μ*m can be phase-matched using type-I BBO with our pump laser, which has a spectral range of 750–860 nm. From the feature of DC-OPA discussed above, the pump and seed pulses should have the same chirp signs. Note that the conversion efficiency from pump to IR pulses will be low because the amplified IR pulses will generate second harmonics, which have a similar phase-matching angle^36^. For BiBO and YCOB crystals, as shown in Fig. 8(b,c), two phase-matching (PM) conditions, indicated by ‘PM1’ and ‘PM2’, can be employed, respectively. When the pump laser has a narrower spectrum bandwidth near 760 nm, PM1 phase-matching, which supports a very broad spectral bandwidth, become the better choice. In fact, a broad spectrum in the range of 1.0–2.0 *μ*m can be expected under a good phase-matching condition^40,41^. Of course, a seed pulse with the optimum chirp should be applied to obtain good conversion efficiency. 100-mJ-class or even joule-class IR pulses with a duration of two cycles can be expected when 1-J- to 10-J-class pump pulses are used.

For our pump laser with a broad spectral bandwidth, PM2 has higher conversion efficiency for generating a broadband signal pulse when the pump and seed pulses have the same chirp sign. The spectrum is sufficiently broad (1.1–1.6 *μ*m) to support a few-cycle pulse duration. It is also found that a BiBO crystal supports a slightly broader phase-matching bandwidth than a YCOB crystal for our pump laser. However, the much larger crystal aperture size of YCOB (80 mm) than that of BiBO (7 mm) makes YCOB a better choice for experiments. In an word, broad spectra supporting a few-cycle pulse duration can be obtained by the DC-OPA method using a variety of nonlinear crystals.

### Further energy scaling of DC-OPA

A typical OPCPA system requires a high-energy pump laser with an optimum pulse duration of 1 ps order to maintain the efficiency and spectral bandwidth^23^. This requirement is very challenging using current laser technology. In fact, the highest energy of a ps pump laser is 1 J with a pulse duration of ~5 ps, which greatly limits the further scaling up of the IR fs pulse energy to the joule class. In contrast, high-energy Ti:sapphire lasers with an output energy range from 1 J^42^ to 100 J order^2,43^ have been developed and utilized for applications. Hence, it is straightforward to futher scale up the output energy of DC-OPA to 1 J^26^ and even 10 J-order by using high-energy Ti:sapphire lasers.

Here, we design experimental parameters for further energy scaling using a BBO crystal under type-II phase matching based on our experiment. Compared with our previous discussions^26^, we present more detailed parameters in this paper. Moreover, we present optimized chirps for seed and pump pulses, which are important for obtaining high conversion efficiency and a broad spectrum during energy scaling in DC-OPA.

The aperture size of the BBO crystal used is 22 mm × 22 mm, which is commercially available. The pump laser is a Ti:sapphire laser with a pulse energy of 15 J^44^ (such a laser system is also commercially available, e.g., Alpha XS 1 PW from Thales, France) and a TL pulse duration of shorter than 40 fs, and the pump beam diameters are 6 mm and 20 mm with pulse energies of 1.4 J and 13.6 J for the first and second stages, respectively. Note that a pump laser with a longer TL duration (narrower spectral bandwidth) can provide similar output efficiency for the DC-OPA system. However, the spectral bandwidth of the IR pulses will be narrower (longer TL pulse duration) in this design. Of course, the design parameters depend on the desired specifications of the IR pulses. The seed pulses have the same beam size. Under these conditions, the pump intensity in the first and second stages is ~6 GW/cm^2^ with ~550 ps pulse duration, which is below the damage threshold of the BBO crystal as estimated using the square-root scaling law^20^. The pump pulse is positively chirped with GDD and TOD values of 3.5 × 10^6^ fs^2^ and −6.5 × 10^6^ fs^3^, respectively. Simultaneously, the seed pulse with a pulse energy of ~10 *μ*J is temporally stretched to the same pulse duration of ~550 ps using an Öffner stretcher. The Öffner stretcher can provide a dispersion with GDD and TOD values of ~8 × 10^6^ fs^2^ and −1 × 10^8^ fs^3^, respectively, which enable the chirp for the seed pulse to achieve an almost perfect phase-matching condition. More details are presented in the Supplementary Information (see Supplementary S-Fig. 3 and discussion). To achieve sufficient gain, the thicknesses of the crystals are 15 mm and 9 mm in the first and second stages, respectively. The temporal walk-offs between the pump, signal, and idler pulses are on the order of 1% of their pulse duration and thus can be neglected. The expected total output energy after DC-OPA is approximately 4.5 J with 30% conversion efficiency, which is estimated from the results of our experiment. The high-energy signal pulse is compressed to a duration of about 40 fs using a grating compressor. Thus, the peak power approaches 100 TW. Further energy scaling using a BBO crystal is also possible if a pump laser with even higher energy is employed by further increasing both the pump and seed pulse durations.

A YCOB crystal instead of BBO will be suitable for further energy scaling. There are three main considerations. First, a very large YCOB crystal with a size of up to *ϕ*80 × 200 mm^3^ can be fabricated^41,45^, which will be convenient for experiments on further energy scaling. Second, a type-I YCOB crystal supports a broader phase-matching bandwidth than a type-II BBO crystal, as shown in Figs 3(c) and 8(c). Thus, it can be utilized to generate high-energy IR pulses with few-cycle pulse durations. Third, a YCOB crystal has a high laser damage threshold comparable to that of a BBO crystal. Using a large YCOB crystal as a nonlinear crystal for the DC-OPA system, a 5 PW Ti:sapphire laser^2^ with a pulse energy of ~150 J can be employed to pump the DC-OPA system by increasing the pump beam size to 24 mm and 80 mm in the first and second stages, respectively. The pump pulse duration is ~550 ps which is the same as above. A seed pulse with a optimum chirp with GDD and TOD values of 3.1 × 10^6^ fs^2^ and −5.2 × 10^6^ fs^3^, respectively, is also provided by an Öffner stretcher. More details of the calculation and a discussion can be found in the Supplementary Information (see Supplementary S-Fig. 4 and discussion). The phase-matched bandwidth is sufficient to generate few-cycle IR pulses. Thus, IR pulses with ~45 J energy and few-cycle duration will be obtained using the DC-OPA method. The peak power reaches the PW level.

### Characteristics of standard OPA, OPCPA, FOPA, and DC-OPA

Strictly speaking, OPCPA and DC-OPA are different types of OPA. Here, we consider another OPA method called frequency domain OPA (FOPA)^46^. FOPA is derived from OPA and has the ability to generate high-energy few-cycle IR laser pulses. To help readers understand the different techniques, the characteristics of standard OPA, OPCPA, FOPA, and DC-OPA are shown in Table 1, which are based on previous studies. OPA, FOPA, and DC-OPA employ broadband femtosecond lasers as pump sources, while OPCPA employs a narrow-band laser as a pump (Table 1 lists the commonly employed pump lasers). The energy-scaling capability of standard OPA is determined by the dimension scaling of nonlinear crystals. OPCPA, FOPA, and DC-OPA can scale up the output energy by employing pump pulse with long durations, and thus are not restricted by the manufacturing capability of crystals. Compared with OPCPA and FOPA, DC-OPA has higher efficiency, achieving an IR femtosecond laser energy in the 100 mJ class in an experiment for the first time. For OPA, OPCPA, and DC-OPA, the signal (amplified seed) and idler (DFG between pump and seed) pulses can be obtained as output pulses and utilized for applications. However, for FOPA, idler pulses are difficult to use because they cannot be recovered spatially and compressed temporally by the grating, which is only designed for the signal pulses. Table 1 also lists the carrier-envelope phase (CEP) stability of the output pulses when seeded by a seed pulse with a stable CEP. OPCPA and DC-OPA can maintain the same CEP stability as that of the standard OPA if bulk and chirp mirror compressors are employed. In contrast, FOPA tends to deteriorate the CEP stability owing to the employment of gratings, which are key elements in FOPA but one of the main noise sources for the CEP^27,47^. In addition, the spectrum bandwidth and efficiency after FOPA are strongly determined by the gratings, which are very difficult to fabricate with high efficiency over a broad spectrum (e.g., higher than 90% over a spectral range of one octave). To efficiently generate IR pulses with a broad spectral bandwidth, OPA and OPCPA require thin crystals with a broad phase-matched bandwidth. FOPA and DC-OPA each have their own options given as below. By increasing the number of crystals in the Fourier plane (spatial), which are tuned to be phase-matched at different wavelengths, a broad spectrum can be obtained in FOPA. For DC-OPA, by optimizing the temporal chirps of pump and seed pulses, a broad spectrum with high efficiency can be obtained, which does not suffer a loss of spectrum bandwidth when thicker crystals are used to increase the efficiency of energy scaling. Considering all the aspects listed in Table 1, it can be concluded that DC-OPA is a superior method for generating high-energy IR fs pulses with high efficiency and excellent energy-scaling ability.

## Methods

### Experimental setup

**Figure 9:**
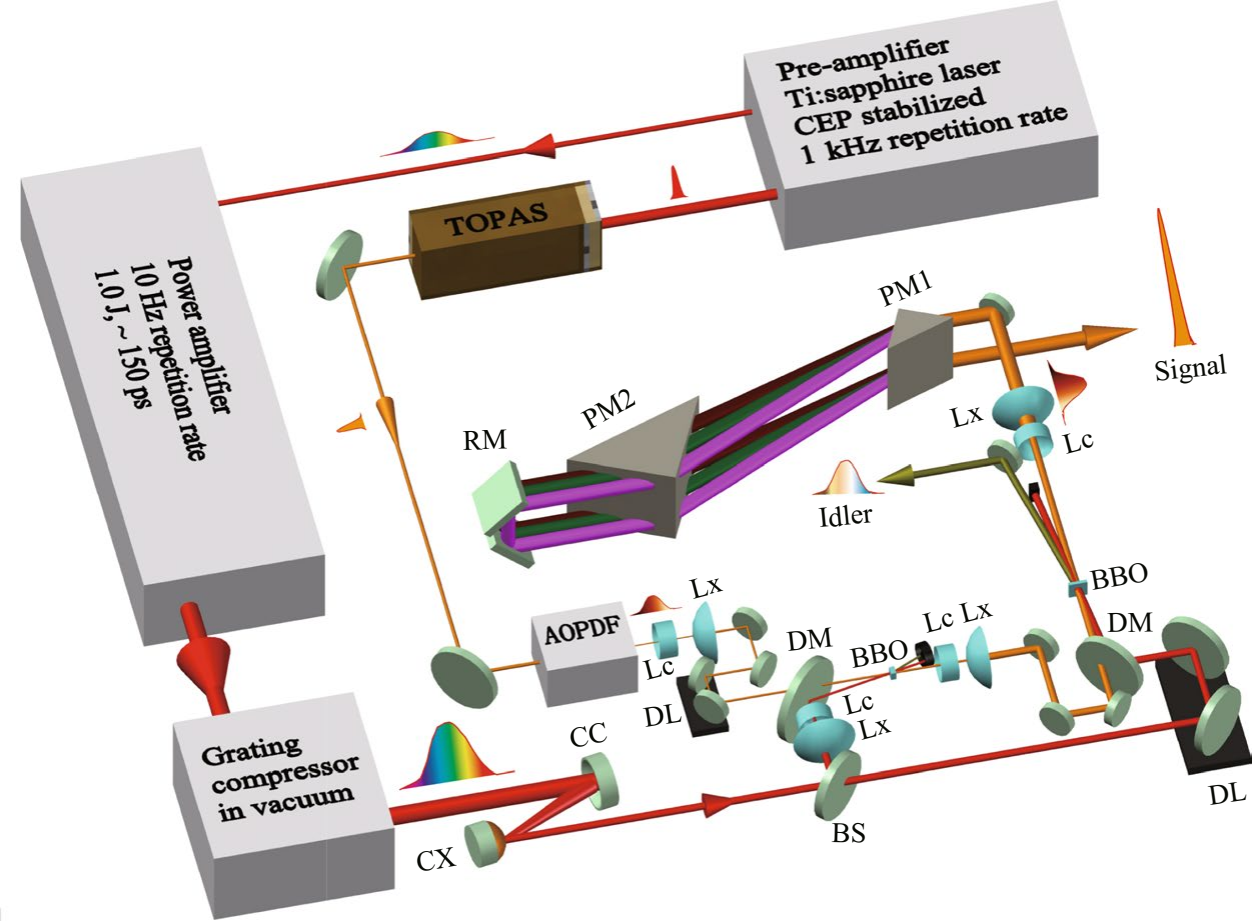
Schematic of the experimental setup. PM: prism (SK1300); BS: beam splitter; DM: dichroic mirror; DL: delay line; Lc: concave lens; Lx: convex lens; CX: convex mirror; CC: concave mirror; RM: roof mirror.

The experimental setup is shown in Fig. 9. The laser system starts from a pre-amplifier (Legend Elite Duo, Ti:sapphire CPA from Coherent Inc.), which has a central wavelength of ~805 nm and a repetition rate of 1 kHz. Before the laser beam is compressed by the compressor inside the pre-amplifier, it is split into two beams by a beam splitter. The uncompressed beam with a pulse energy of 1.5 mJ and a pulse duration of ~150 ps, whose beam pointing is actively stabilized by a beam stabilizer (Aligna 4D, TEM Messtechnik), is further amplified to a pulse energy of 1 J by a multipass power amplifier (MPA). The MPA is pumped by two flash-lamp-pumped lasers (Precision-II, Continuum) which are operated at a 10 Hz repetition rate. After the MPA, a grating compressor, which is built inside a vacuum chamber, is employed to manipulate the pulse duration as well as the temporal chirp. Then these pulses with an energy of 700 mJ and a beam diameter of 20 mm are utilized to pump a two-stage DC-OPA system. Another beam from the pre-amplifier is compressed to a 25 fs pulse and an energy of 3–4 mJ by a grating compressor. This pulse is employed to pump an OPA system (TOPAS Prime, Coherent Inc.), which provides wavelength-tunable IR pulses in the range of 1.2–1.6 *μ*m. An IR pulse with an energy of ~15 *μ*J and a beam size of ~2.5 mm passes through an AOPDF (Dazzler, Fastlite), which is programmed to increase the pulse duration with a precisely manipulated temporal chirp. Then, the temporally chirped IR pulse with a pulse energy of ~2 *μ*J is employed as a seed pulse for the DC-OPA system. The beam diameter of seed pulses is adjusted to match that of the pump beam using concave and convex lens pairs. Type-II BBO crystals (*θ* = 27°) without anti-reflection coatings are employed as nonlinear crystals in both stages. The crystal dimensions are 10 × 10 × 5 mm^3^ and 20 × 20 × 3 mm^3^ for the first and second stages, respectively. In the first stage, the pumping energy is ~70 mJ, corresponding to a laser intensity of approximately 60 GW/cm^2^ when the pulse duration is ~3.5 ps. A non-collinear configuration (3–6.5° depending on the central wavelength) between the pump and seed is employed for two main reasons. One is that the signal, idler and residual pump pulses can be easily separated in space. The other is that signal pulses with a broader spectrum bandwidth might be obtained under a non-collinear configuration. In the first stage, the parametric amplification gain is 2000–5000. Only the signal pulse is selected and sent to the second stage of the DC-OPA system for further amplification. The second stage is also constructed in a non-collinear configuration with a small angle of ~1° between the pump and signal pulses. The purpose of this configuration is to separate high-energy pump, signal, and idler pulses, which are not obviously helpful for increasing the phase-matched bandwidth. The pumping energy is ~630 mJ corresponding to a laser intensity of approximately 50 GW/cm^2^ on the BBO crystal when the pulse duration is ~3.5 ps. The amplification gain in the second stage is 20–30. The high-energy signal pulse with a beam diameter of ~50 mm is sent to a Brewster angle cut prism compressor (SK 1300) with a throughput of over 85%. The pulse duration and spectral phase of the compressed signal pulse are characterized by the SPIDER method.

## Electronic supplementary material

Supplementary Information
