# Supplementary material for: Towards a petawatt-class few-cycle infrared laser system via dual-chirped optical parametric amplification
Source: Sci Rep. 2018 May 16;8:7692. doi: 10.1038/s41598-018-25783-0 (PMC5955956; doi:10.1038/s41598-018-25783-0)
Supplement: Supplementary file 1 — Supplementary Information [file 41598_2018_25783_MOESM1_ESM.doc]

**Towards a petawatt-class few-cycle infrared laser system via dual-chirped optical parametric amplification**

Yuxi Fu^*^, Katsumi Midorikawa, and Eiji J. Takahashi^†^

Extreme Photonics Research Group, RIKEN Center for Advanced Photonics, RIKEN, 2-1 Hirosawa, Wako, Saitama 351-0198, Japan

*^*^* yxfu@riken.jp

^†^ ejtak@riken.jp

1. **Discussion of effects of GDD and TOD on phase matching**


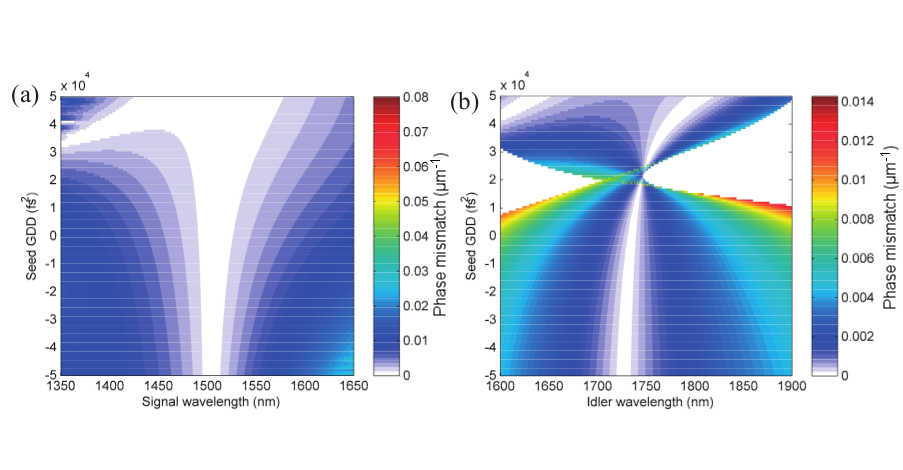


**S-Fig. 1.** Calculation of instantaneous phase mismatch between temporally chirped pump and seed pulses under different GDD values of the seed pulse. GDD and TOD of the pump pulse are fixed at approximately 25000 fs^2^ and -48000 fs^3^, respectively. The TOD for the seed pulse is fixed at ~ 0 fs^3^. The phase-mismatch with absolute values (units of color bar: μm^-1^) are plotted as two-dimensional figures for the signal (a) and idler (b) pulses. The white region in the figure indicates a nearly perfect phase matching condition.

When the seed pulse is temporally stretched under different GDD values (different chirp rates in the temporal domain under different GDD values), different wavelengths across the signal spectrum (GDD is the same as for the seed pulse) are no longer synchronized in time but spread over different time slices. Thus, different wavelengths of the seed pulse can be temporally overlapped with different wavelengths of the pump pulse which is also temporally stretched. Then, we calculate the instantaneous phase mismatch (a small phase-mismatch indicates a better phase-matching condition) between different wavelengths of seed and pump pulses, which are overlapped at different time slices. The calculation result is shown in S-Fig. 1, which shows plots for different signal pulse wavelengths in (a) and for different idler pulse wavelengths in (b). For the signal pulse, it is clear that a broader phase-matched bandwidth over the spectrum is achieved when the GDD is positive, which is the same sign as the GDD of the pump pulse. Thus, a higher conversion efficiency can be obtained under the same GDD value but with a positive sign. For the idler pulse, the spectrum bandwidth is narrowest near 26000 fs^2^, which is similar to the experimental data. Away from this GDD value, the phase-matched spectral bandwidth is broader. However, the group velocity mismatch (GVM) plays a critical role when the seed pulse duration is short near GDD values of 0 fs^2^, which is shown by the experimental result in Fig. 4(b) in the main text.

We also calculate the phase mismatch under different TOD values for the seed pulse as shown in S-Fig. 2. For the signal pulse, as shown in S-Fig. 2(a), different TOD values of the seed pulse do not obviously change the phase-matching conditions within the tuning range of -2×10^5^ fs^3^ to 2.5×10^5^ fs^3^. For the idler pulse shown in S-Fig. 2(b), there is an observable change. When the TOD value of the seed pulse is negative (same as for the pump pulse), the spectral bandwidth with a good phase-match is slightly broader. However, this effect is much smaller than that of the GDD. However, there is an interesting phenomenon that two phase-mismatch values overlap at some of the wavelengths when the TOD of the seed pulse is higher than 50000 fs^3^ and smaller than -150000 fs^3^. This is because that idler pulses with the same wavelength are generated at different time slices when the TOD of the seed pulse is larger than 50000 fs^3^ or smaller than -150000 fs^3^. This effect might result in double pulses. However, as shown in S-Fig. 2(b), the phase mismatch values are different by several orders. Thus, only one pulse will be prominent owing to the much better phase-matching condition. However, in the optimization of DC-OPA, such an effect has to be considered, especially when seed and pump pulses have similar chirp rates. In this experiment, this issue does not arise for the idler pulse when generating 100-mJ-class pulses. This is because the TOD of the seed pulse is much larger than -150000 fs^3^ and has the same sign as that of the pump pulse.


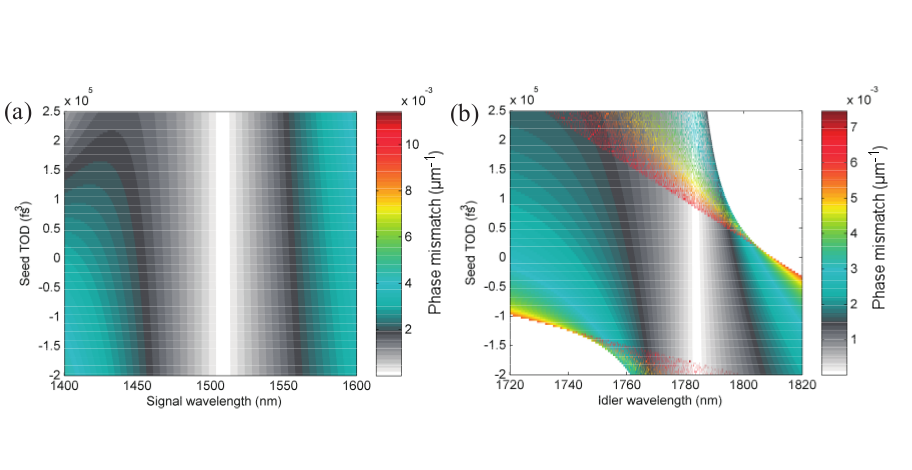


**S-Fig. 2.** Calculation of instantaneous phase mismatch between temporally chirped pump and seed pulses for different TOD values of the seed pulse. GDD and TOD for the pump pulse are fixed at approximately 25000 fs^2^ and -48000 fs^3^, respectively. The GDD for the seed pulse is fixed at 36000 fs^2^. The phase-mismatch with absolute values (units of color bar: μm^-1^) are plotted as two-dimensional figures for the signal (a) and idler (b) pulses. The white region in the figure indicates a nearly perfect phase-matching condition.

1. **Optimum phase matching for energy scaling to approach 100 TW class and PW class using type-II BBO and type-I YCOB crystals**

Here, we calculate optimum phase-matching conditions for the further energy scaling of IR pulses to approach the 100 TW class and PW class. The gratings for controlling the pump pulses have a grooves density of 1480 grooves/mm and an incident angle of 53.87^o^ in this calculation. Of course, gratings with other specifications can also be employed.

A pump pulse energy of 15 J with a transform-limited duration of ~ 23 fs, a central wavelength near 805 nm, and a beam diameter of 22 mm is employed for pumping the DC-OPA system in this calculation. BBO crystals with type-II cutting (θ = 27.85^o^) are employed. We employ an Öffner stretcher having a grating with a groove density of 700 grooves/mm to stretch the seed pulse. For an incident angle of 10^o^ and a grating insertion of 170 mm, we calculate the temporal chirp of the seed pulse after the Öffner stretcher. Then we temporally overlap the chirped seed and pump pulses. The calculated result is shown in S-Fig. 3. The red solid curve shows the wavelengths of the overlapping pump and seed pulses, which are almost the same as the nearly perfect phase-matching condition shown by the white region in the two-dimensional plot. Note that the best phase matching is realized using only an Öffner stretcher. In this design configuration, we do not require an AOPDF to optimize the phase-matching condition. A high conversion efficiency of over 30% (estimated from the results of the experiment in this paper) can be expected for the DC-OPA system. A total pulse energy of the signal and idler pulses of over 4.5 J is also expected. The spectral bandwidth can be preserved, similarly to our experiment in this paper. By compressing the IR pulses using a grating compressor (700 grooves/mm), a pulse with an energy of ~2.0 J (assuming that the grating compressor has an 80% throughput) near 1.5 μm and a duration of ~ 40 fs (FWHM) is expected, which indicates a peak power of ~ 50 TW.


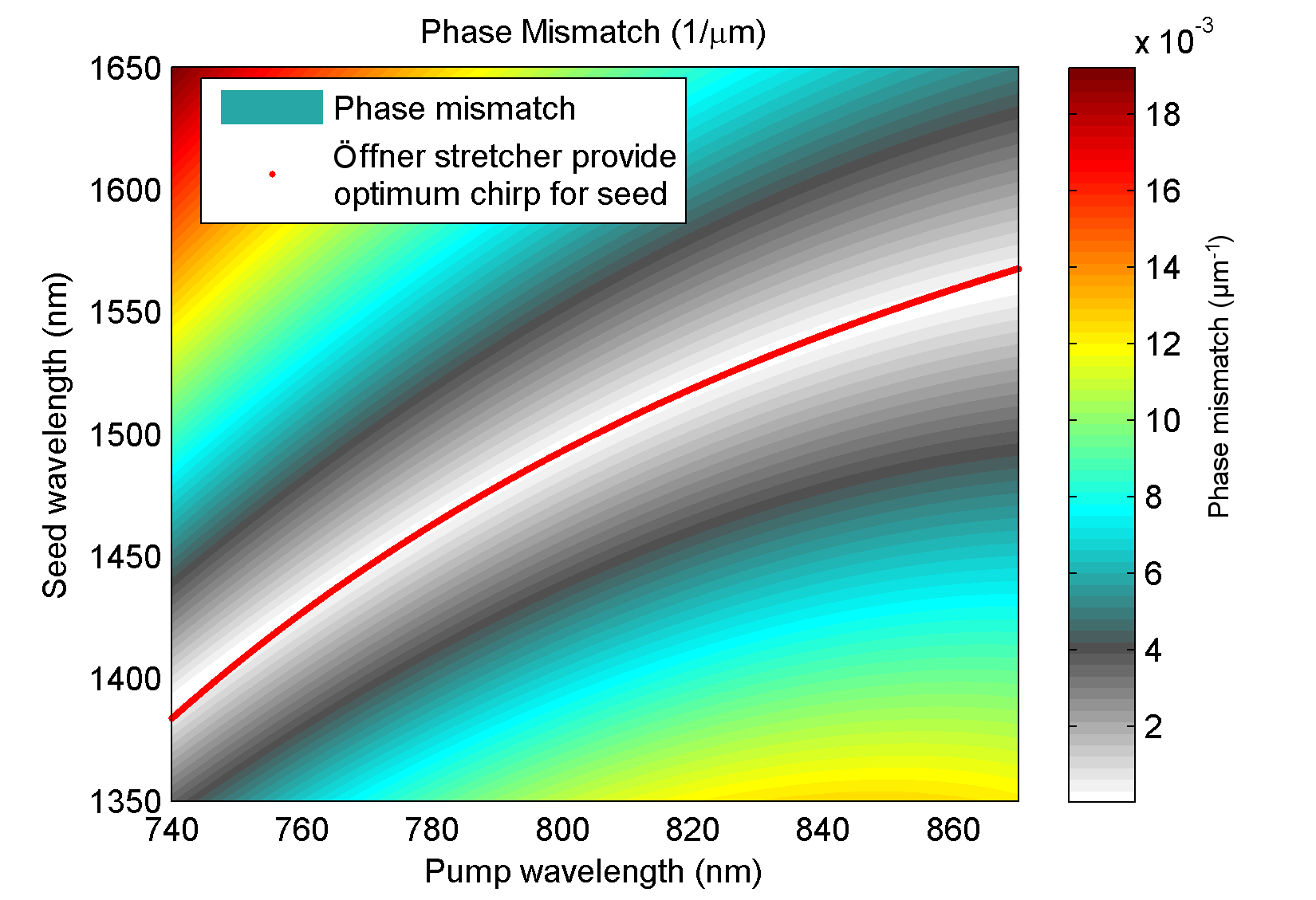


**S-Fig. 3.** Phase mismatch (absolute values) between different wavelength components of signal and pump pulses. The white region in the two-dimensional figure (units of color bar: μm^-1^) indicates a nearly perfect phase-matching condition. The red solid curve indicates the wavelengths of the temporally overlapping pump and seed pulses. The chirp of the seed pulse is provided by an Öffner stretcher.

We choose YCOB crystals under type-I phase-matching cutting (θ = 39.98^o^) for further energy scaling. The Öffner stretcher has a grating with a groove density of 600 grooves/mm for stretching the seed pulse. The incident angle is 70^o^ and the grating insertion is 600 mm. The pump pulse is also the same as above but the energy and beam diameter are increased to 150 J and 80 mm respectively. Similarly to above, we can obtain nearly perfect phase matching as shown in S-Fig. 4. A total output energy of over 45 J can be expected after the DC-OPA system (crystal thicknesses of 20 mm and 12 mm for the first and second stages, respectively). After the grating compressor, a pulse energy of ~ 23 J at 1.25 μm can be obtained. Since a broad spectrum of ~ 1.1–1.4 μm can be phase-matched, as shown in S-Fig. 4, the signal pulse at 1.25 μm can be compressed to a few-cycle duration (estimated to be ~ 15 fs assuming a spectrum with a Gaussian profile). Thus, a PW-class IR pulse with a peak power of ~ 1.5 PW can be expected.


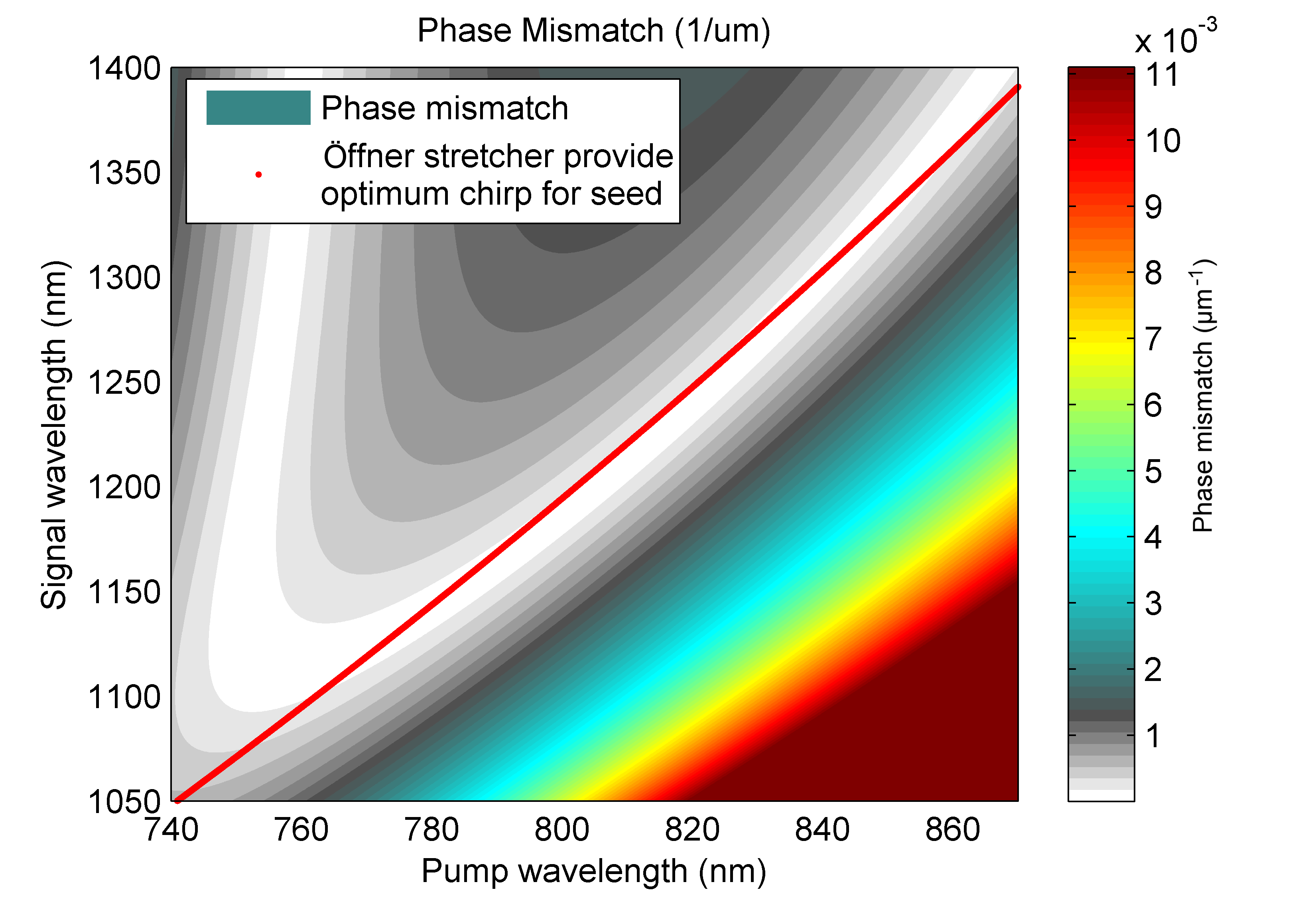


**S-Fig. 4.** Phase mismatch (absolute values) between different wavelengths of signal and pump pulses. The white region in the two-dimensional figure (units of color bar: μm^-1^) indicates a near to perfect phase-match condition. The red solid curve indicates the wavelengths of the temporally overlapping pump and seed pulses. The chirp of the seed pulse is provided by an Öffner stretcher.
